# *Streptococcus mutans* isolated from a 4‐year‐old girl diagnosed with infective endocarditis

**DOI:** 10.1002/cre2.220

**Published:** 2019-07-22

**Authors:** Yoshio Kondo, Tomonori Hoshino, Midori Ogawa, Kiyoshi Hidaka, Tomoyuki Hasuwa, Hiroyuki Moriuchi, Taku Fujiwara

**Affiliations:** ^1^ Department of Paediatric Dentistry Nagasaki University Graduate School of Biomedical Sciences Nagasaki Japan; ^2^ Department of Paediatric Dentistry Meikai University School of Dentistry Saitama Japan; ^3^ Department of Microbiology, School of Medicine University of Occupational and Environmental Health Japan Kitakyushu Japan; ^4^ Department of Paediatrics Nagasaki University Graduate School of Biochemical Sciences Nagasaki Japan

**Keywords:** biofilm, infective endocarditis, *Streptococcus mutans*

## Abstract

**Objectives:**

Infective endocarditis (IE) has an extremely high fatality rate. In this study, we isolated a strain of *Streptococcus mutans*, which we called HM, from the blood drawn from a 4‐year‐old girl diagnosed with IE. We aimed to fully type the HM strain and investigate its biological properties, including its virulence with respect to IE.

**Material and methods:**

A 16S rRNA phylogenetic tree and glucosyltransferase gene sequences were used to type HM. Serotyping was performed using the Ouchterlony method. Morphological observations were made using phase contrast and electron microscopy. Fibrinogen adhesion and biofilm formation were investigated to examine the tissue colonization properties of HM, whereas its bodily origin was determined from its fingerprinting pattern.

**Results:**

The isolated strain was *S. mutans* serotype *e.* However, its morphology was observed to be short chains, unlike that of the NCTC 10449 reference strain. Fibrinogen adhesion and biofilm formation were more apparent than in NCTC 10449. The fingerprinting pattern showed that HM came from the patient's saliva.

**Conclusions:**

HM differs from NCTC 10449 in its higher fibrinogen affinity. HM was also found to be derived from the oral cavity. These results highlight the importance of good oral hygiene for the prevention of IE in children.

## 1 INTRODUCTION

Dental antibiotic prophylaxis for prevention of infective endocarditis (IE) has been recommended by some guidelines since the 1950s. However, there is no strong evidence to support this practice. In addition, concerns regarding the development of allergies and the emergence of resistant bacteria caused by the administration of antimicrobial agents have also been expressed. Based on these issues, since the 2000s, a review of antimicrobial administration at the time of dental treatment has been conducted in Western countries (Danchin, Duval, & Leport, 2005; Excellence NIfHaC, 2008; Habib et al., 2009; Wilson et al., 2007). In the guidelines of France in 2002 (Danchin et al., 2005), those of the United States in 2007 (Wilson et al., 2007), and those of Europe in 2009 (Habib et al., 2009), the administration of antibiotics for moderate‐risk patients was not recommended, whereas the administration of antibiotics for high‐risk patients was recommended. Moreover, the National Institute for Health and Care Excellence guidelines of the United Kingdom in 2008 made a recommendation to not use preventative antibiotics in all patients, including those in the high‐risk group (Excellence NIfHaC, 2008).

After changing the guidelines, the number of preventative antibiotic administrations has dropped sharply in the United Kingdom (Dayer et al., 2015; Thornhill et al., 2011). A slight but statistically significant increase in the number of IE episodes was observed after 5 years (Dayer et al., 2015). Regarding the U.S. and European countries except the United Kingdom, there are various reports about the increase or decrease in the incidence of IE (Bikdeli et al., 2013; Cahill et al., 2017; DeSimone et al., 2015; Duval et al., 2012; Erichsen, Gislason, & Bruun, 2016; Keller et al., 2017; Mackie, Liu, Savu, Marelli, & Kaul, 2016; Pant, Deshmukh, & Mehta, 2015; Toyoda et al., 2017). Among these reports, the number of IE episodes was reported to have increased due to oral streptococci, suggesting a causal relationship between preventive medication and dental treatment (Pant et al., 2015). Although much controversy about preventive administration at the time of dental treatment remains, IE has serious consequences once it occurs and has a great impact on patients' lives (Franklin et al., 2016). Therefore, it is important to prevent IE, and in addition to prophylaxis with antibiotics, education of patients in the maintenance of oral hygiene is necessary.

IE is most frequently caused by staphylococci or streptococci bacteria (Hall‐Stoodley, Costerton, & Stoodley, 2004; Moreillon & Que, 2004; Murdoch et al., 2009). Population‐based cohort studies show that viridans group streptococci are the most common IE‐causing organisms, followed by *Staphylococcus aureus* (Tleyjeh et al., 2005). Viridans group streptococci constitute the largest group among the streptococci, which are also known to be the most prevalent bacterial group in the oral cavity. Within the viridans streptococcal group, *Streptococcus sanguinis* and *Streptococcus oralis* are the pathogens most frequently isolated from patients with IE. *Streptococcus mutans*, a well‐known cariogenic bacterium, also belongs to the viridans streptococcal group. Although this bacterium has also been reported as an IE‐causing pathogen, its detection frequency is very low (Douglas, Heath, Hampton, & Preston, 1993). Therefore, we analyzed the genetic and biological characteristics of the bacterial strain we isolated from a 4‐year‐old girl diagnosed with IE and investigated its relationship with IE.

## 2 MATERIALS AND METHODS

### 2.1 Subject

The patient was a 4‐year‐old girl with a ventricular septal defect (with no history of surgery) who visited Nagasaki University Hospital, Japan, with a fever of unknown origin. The fever appeared approximately 2 months before her visit. She had received clarithromycin and cefcapene pivoxil as oral antimicrobial drugs, which had an antipyretic effect. However, when the oral dosing was interrupted, the pyrexia returned. The echocardiography examination showed vegetation near the apex of the tricuspid valve. Gram‐positive cocci were detected by blood culture testing. On the basis of these findings, she was diagnosed with IE according to the Duke diagnostic criteria. For treatment, based on the sensitivity profile revealed by the antibiotic test results, ampicillin was selected and infused intravenously. An antipyretic effect was observed the next day, and antibiotic therapy was administered for a total of 4 weeks. Blood culture tests after the antimicrobial therapy were negative, and the patient remained apyrexial. After discharge, she visited pediatric dentistry; a few dental caries were observed, and poor oral hygiene was evident.

### 2.2 Bacterial strains

The bacterial strain isolated from the patient's blood sample, designated here as strain HM, was stored at −70°C until use. *S. mutans* NCTC 10449 was used as the reference strain (Hardie & Genus Streptococcus, 1986; Smibert et al., 1994). Both strains were grown anaerobically in brain heart infusion (BHI) broth (Difco Laboratories) and on trypticase soy agar plates (Difco Laboratories) supplemented with defibrinated sheep blood (5% vol/vol).

### 2.3 Determining the species isolated from the specimen

A phylogenetic tree based on 16S rRNA was constructed using MEGA (ver. 7.0) by the neighbor‐joining method. The 16S rRNA DNA sequences from the data published for oral streptococci that were used in this study are as follows: *Streptococcus mitis* NCTC 12261 (GenBank accession D38482.1), *Streptococcus pneumoniae* NCTC 7465 (X58312.1), *S. oralis* CCUG 24891 (DQ303190.1), *Streptococcus gordonii* NCTC 7865 (D38483.1), *S. sanguinis* JCM 5708 (AB596946.1), *Staphylococcus intermedius* SK54 (JN787124.1), *Streptococcus constellatus* ATCC 27823 (Z69041.1), *Streptococcus anginosus* ATCC 33397 (Z69038.1), *Streptococcus pyogenesis* ATCC 12344 (AB002521.1), *Streptococcus bovis* ATCC 33317 (M58835.1), *Streptococcus vestibularis* CCRI 17387 (FJ154805.1), *Streptococcus salivarius* CCUG 7215 (FJ154803.1), *Streptococcus sobrinus* ATCC 33478 (AY188349.1), *S. mutans* NCTC 10449 (X58303.1), and our own previously determined database sequence for the *S. mutans* HM strain (NZ_BDOS00000000.1; Kondo et al., 2017).

### 2.4 Scanning electron microscopy observations

*S. mutans* strains were precultured on BHI agar for 24 hr. Bacterial cells were suspended in phosphate buffered saline (PBS). They were fixed with 2.5% glutaraldehyde in PBS overnight at 4°C, washed with PBS, postfixed with 1% osmium tetroxide in PBS for 2 hr at room temperature, and washed with double distilled water. The fixed specimens were immersed in t‐butyl alcohol after dehydration via a graded series of acetone and then freeze dried. They were coated with osmium and observed using a Hitachi S‐4500 scanning electron microscope.

### 2.5 Fibrinogen‐binding and biofilm formation assays

The fibrinogen‐binding properties of the *S. mutans* strains were evaluated according to the methods described previously (O'Toole, 2011), with some modifications. Tissue culture plates (48‐well, Becton Dickinson) were coated with fibrinogen (Sigma‐Aldrich Co.) prepared in carbonate–bicarbonate buffer (0.05‐M Na_2_CO_3_, pH 9.6) and then incubated overnight at 4°C. The plates were then washed three times with PBS and blocked for 1.5 hr with bovine serum albumin (Sigma‐Aldrich Co.) in PBS at 37°C. BHI broth (1 ml) and 1 μl of bacterial cells from an overnight culture of *S. mutans* were added to each well. After 24 hr of incubation at 37°C, the adherent cells were washed three times with PBS, stained with 100 μl of 0.1% crystal violet in water for 10 min, and washed three times with PBS. The dye was dissolved by the addition of 30% acetic acid (100 μl) before the optical density for each strain at 570 nm was determined. The data are expressed as the mean and standard deviation of six independent experiments with four wells per sample.

## 3 RESULTS

**Figure 1:**
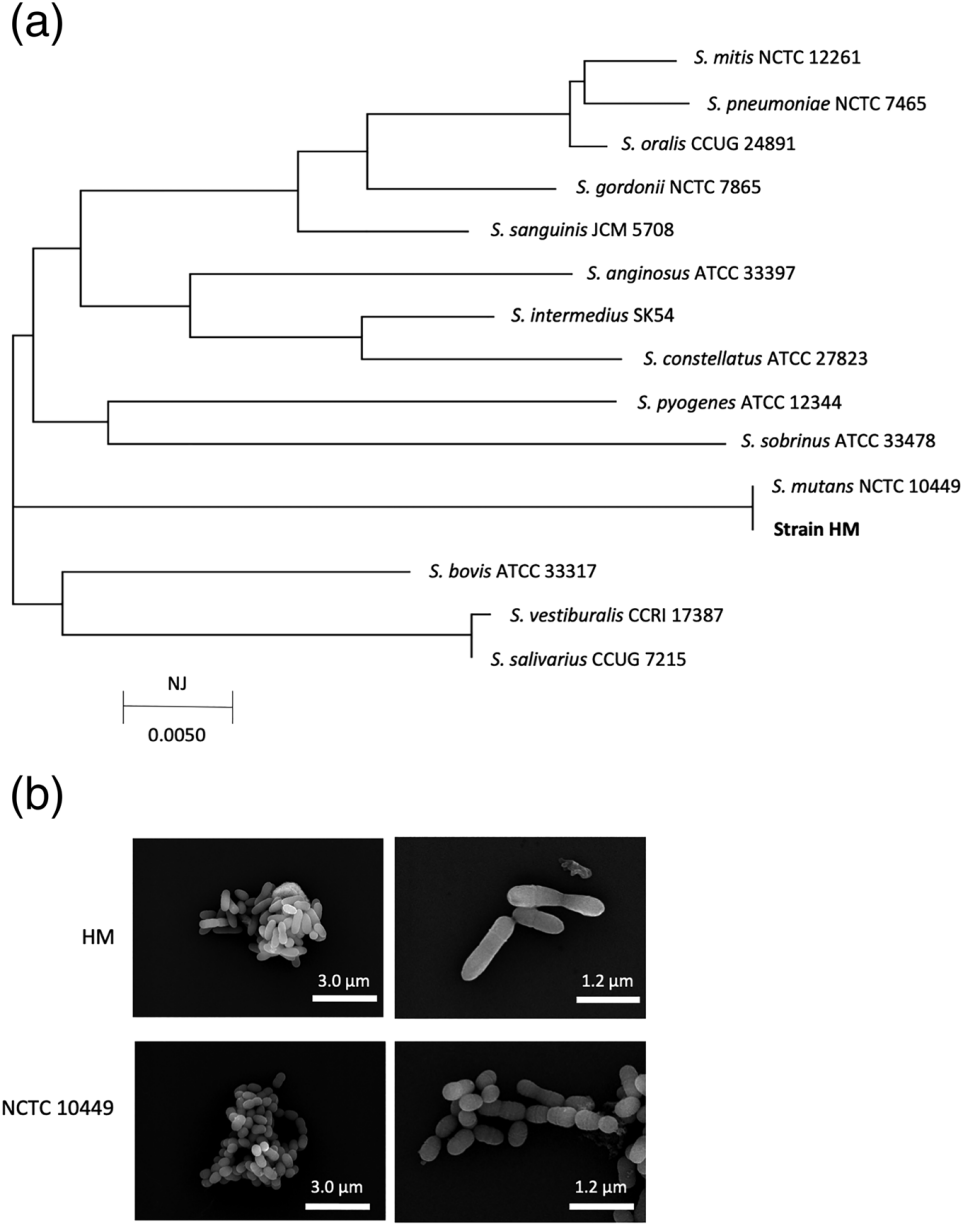
Bacterial identification and microscopic observations. (a) The phylogenetic tree, which was based on the 16S rRNA gene, was constructed by the neighbor‐joining (NJ) method. (b) Scanning electron microscopy observations of Streptococcus mutans HM and NCTC 10449 strains

The bacterial strain isolated from the blood drawn from the 4‐year‐old girl who was hospitalized for IE was designated as HM. To identify the bacterial species, a phylogenetic tree based on 16S rRNA was constructed, and it showed that the HM strain displayed high sequence homology with *S. mutans* NCTC 10449 (Figure 1a). However, the optical microscopic observations revealed that HM displayed a shorter chain‐like structure than that of the NCTC 10449 strain. After further examination with an electron microscope, the individual cells of the HM strain were found to be elongated, similar to bacteria in the Corynebacterium genus, and they were also club shaped (Figure 1b).

**Figure 2:**
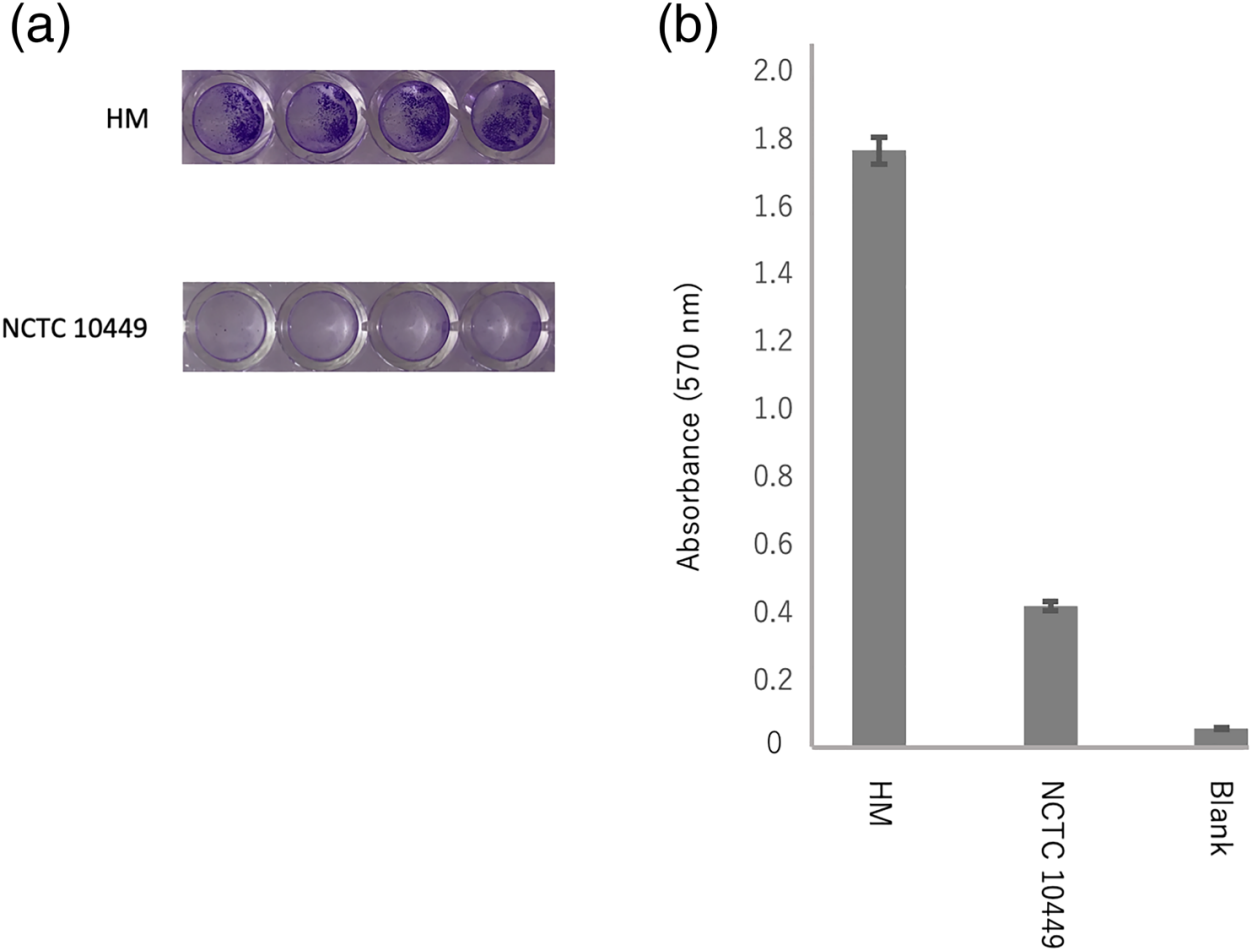
Adhesion to fibrinogen and biofilm formation in the Streptococcus mutans HM strain. (a) Bacterial cells were grown for 24 hr in brain heart infusion broth in a 48‐well plate containing immobilized fibrinogen, and biofilm formation was assessed using a crystal violet‐based assay. (b) Bars represent the average absorbance values at 570 nm, with four wells used for each bacterial strain. The assay was performed six times. “Blank” represents the experiment when no bacterial culture was added

Platelet aggregation, which can occur after infection with pathogenic bacteria, is thought to be one of the most important factors affecting the pathogenesis of IE. Hence, in this study, we also investigated fibrinogen adhesion and biofilm formation. In both respects, strain HM exhibited higher levels than NCTC 10449 (Figure 2).

## 4 DISCUSSION

This is a report of *S. mutans* isolated from a 4‐year‐old girl diagnosed with IE in Japan. To characterize the bacterium responsible for the IE, we conducted a phylogenetic tree analysis based on 16S rRNA sequences for HM and various database strains. The phylogenetic tree showed that the HM strain shared high sequence homology with *S. mutans.* Although 16S rRNA sequence analysis is a widely used method for typing bacteria, we also used an additional sequence data analysis for discrimination purposes because the sequence homology among oral streptococcal species is relatively high (Bentley, Leigh, & Collins, 1991). Further analysis using the glycosyltransferase gene sequence, as previously reported (Hoshino et al., 2004), also identified the HM strain as an *S. mutans* member (data not shown). Also, we classified the serotype of HM, which involved extracting the cell surface polysaccharide molecules and observing their reaction with a specific antibody. The results showed that HM was serotype *e.* *S. mutans* is classified into three serotypes, namely, *c*, *e*, and *f*, based on the structures of the cell wall‐associated polysaccharides (Okahashi, Koga, Akada, & Hamada, 1983). In general, the major serotype found in the oral cavity is serotype *c* (approximately 70–80% of isolates), followed by serotype *e* (approximately 20%), whereas the prevalence of serotype *f* is low (Fitzgerald, Fitzgerald, Adams, & Morhart, 1983; Hamada, Masuda, & Kotani, 1980; Hirasawa & Takada, 2003; Shibata et al., 2003). Nonserotype *c* strains of *S. mutans*, namely, serotypes *e* and *f*, have been detected at high frequencies in specimens from patients who underwent surgery for the removal of atheromatous plaques and heart valve replacement (Nakano et al., 2007). It has been speculated that nonserotype *c* strains are isolated at higher frequencies than other strains because of their prolonged persistence in the blood (Nakano et al., 2007). It was also reported that strains of serotypes *e* and *f* can invade primary human coronary artery endothelial cells (Abranches et al., 2011). Invasive strains were also found to be significantly more virulent than noninvasive strains in the *Galleria mellonella* (greater wax worm) model of systemic disease (Abranches et al., 2011).

Our observation of the HM strain revealed that the chain structure of this bacterium is shorter than that of NCTC 10449. Some studies have predicted that the conditions that may affect cellular chain length may also affect adhesion (Murchison, Larrimore, Hull, & Curtiss, 1982) and aggregation (Murchison et al., 1982; Nakano, Fujita, Nishimura, Nomura, & Ooshima, 2005), both of which can contribute to virulence. Streptococcal cellular chain length and morphology are influenced by several factors. Previous studies have suggested that cell wall components such as peptidoglycan, lipoteichoic acids, and cell wall‐anchored proteins can greatly affect the morphology of the cells (Thibodeau & Ford, 1991). However, the factors affecting the morphology of the HM strain await elucidation.

Platelet aggregation, which occurs after infection with pathogenic bacteria, is thought to be one of the most important factors affecting the pathogenesis of IE. The mechanisms by which endocarditis‐causing bacterial species induce platelet aggregation have also been studied (Douglas, Brown, & Preston, 1990; Herzberg et al., 2005). These studies have reported that bacterial extracellular matrix binding proteins are potential factors affecting platelet aggregation. Regarding *S. mutans*, extracellular matrix binding protein also mediates platelet aggregation and biofilm formation (Bedran, Azelmat, Spolidorio, & Grenier, 2013; Nomura et al., 2014), and the results for HM are consistent with these findings.

It is generally known that *S. mutans* is present on the surface of teeth. Therefore, in this study, to clarify the pathway by which HM invaded the bloodstream, the strains present in the dental plaque specimen from this patient were analyzed. The fingerprinting patterns of the *S. mutans* resident in the oral cavity of this patient and strain HM are concordant (data not shown), making it likely that the HM strain originated from the oral cavity. It is also known that severe tooth decay may lead to a continuous focus of infectious disease, whereby pathogenic bacteria may reach pathogenic lesions. However, recent studies suggest that bacteremia occurs even with routine daily tooth brushing and dental flossing procedures (Forner, Larsen, Kilian, & Holmstrup, 2006; Guntheroth, 1984; Sonbol, Spratt, Roberts, & Lucas, 2009). Additionally, in this case, because severe dental caries were not observed, the origin of HM is likely associated with everyday life, with the IE seemingly having developed from bacteremia. Recently, we have completed the draft genome sequence for the HM strain and deposited it in the DDBJ/EMBL/GenBank database under accession no. BDOS00000000 (Kondo et al., 2017). Having access to the complete genomic sequence and the findings from the present study will be useful for clarifying the pathogenesis of *S. mutans*‐related IE.

### 4.1 Why this paper is important to pediatric dentists

- In this study, it was concluded that minor oral mucosal damage due to everyday life activities, such as teeth brushing, was the cause of IE for the following reasons: (a) The causative microorganisms were oral streptococci, (b) no dental treatment occurred before the onset of IE, and (c) the oral cavity was in an unsanitary condition.
- According to the 2007 IE guidelines from the American Heart Association, maintaining daily oral cleansing decreases bacteremia levels, which is more important for the prevention of IE than the administration of antibiotics prior to dental treatment. Based on this fact, the guidelines also call for regular dental examinations and guidance with regard to the correct oral care for patients. Pediatric dentists have many opportunities to examine high‐risk patients and should bear this in mind.

## CONFLICT OF INTEREST

The authors declare no conflict of interest.

## AUTHOR CONTRIBUTIONS

T. H., K. H., and T. H. have identified the bacterial species. Y. K. carried out the observation with phase contrast microscope and adhesion experiment. M. O. performed the observation with an electron microscope. All authors analyzed the results, contributed to writing the manuscript, and approved the final version of the manuscript.
